# Tit for tat or good for evil? Linking customer incivility, hostility, guilt, and employee behaviors

**DOI:** 10.3389/fpsyg.2022.1053145

**Published:** 2023-01-12

**Authors:** Chong Chen, Mingyu Zhang, Yihua Zhang

**Affiliations:** ^1^School of Economics and Management, Beijing Jiaotong University, Beijing, China; ^2^Graduate School of Education and Psychology, Pepperdine University, Los Angeles, CA, United States

**Keywords:** customer incivility, hostility, guilt, revenge behavior, customer-oriented behavior

## Abstract

The existing literature overemphasizes the negative effects of customer incivility on service employees. However, the positive effects of customer incivility on employee behavior are rarely mentioned. Drawing on affective events theory and attribution theory, we used a moderated dual-mediator causal model to explore the effect of customer incivility on employees’ revenge behavior and customer-oriented behavior through hostility and guilt, and the moderating role of customer blame attribution. An empirical study with a sample of 366 employee-supervisor pairs and two-wave, two-source data indicated that customer incivility positively impacts revenge behavior *via* employees’ hostility, and this relationship is reinforced by customer blame attribution. In contrast, customer incivility positively impacts customer oriented behavior *via* employees’ guilt, and this relationship is weakened by customer blame attribution. This study expanded the literature on customer incivility and emotion, and provided significant practical implications for organization on how to help frontline employees deal with customer incivility.

## Introduction

In employee–customer interactions, employees often encounter unfriendly and impolite treatment from customers (Sliter et al., 2012). In service occupations, about 70% of service employees affected by customer incivility events, and the prevalence of such event, to some extent, explained the high turnover rate in the service industry (Medler-Liraz, 2020; Shin and Hur, 2022). If not managed, such events will harm enterprises’ reputation and their economic returns (Durana et al., 2021; Valaskova et al., 2021). As one of the major negative factors in the service employee work environment, customer incivility has received a lot of attention from many scholars. Customer incivility is considered as a kind of job stressors, which affects employees’ emotions, cognition, attitudes, and behaviors, and can hinder the smooth progress of their work and bring a series of negative outcomes, such as leading to employees’ negative affectivity (Cheng et al., 2020b), emotional exhaustion (Kern and Grandey, 2009; Al-Hawari et al., 2020), burnout (Han et al., 2016), withdraw (Sliter et al., 2012; Boukis et al., 2020), role stress (Boukis et al., 2020), revenge behavior (Bedi and Schat, 2017), employee incivility (Walker et al., 2014), and dysfunctional behavior (Balaji et al., 2020).

Although many previous research has provided theoretical support and empirical evidence for the exploration of the outcome variables of customer incivility, there are still a number of neglected issues that deserve to continue to be explored. Firstly, most studies overemphasize the effects of customer incivility on general emotional states such as negative affectivity, ignoring the experience of specific discrete emotions (Cheng et al., 2020a). Moreover, past research focused on the negative effects of customer incivility, without considering the possibility of its positive impacts. Finally, the exploration of boundary conditions is rarely interpreted from the perspective of attribution (Bedi and Schat, 2017; Cheng et al., 2020b). It is known that differences in attribution will lead to different judgments on the same event (Martinko et al., 2011).

In order to solve these issues, we use discrete emotions (i.e., hostility and guilt) as an important mechanism to explain why customer incivility impact employees’ behaviors (i.e., employees’ revenge behavior and customer-oriented behavior) based on affective events theory (Weiss and Cropanzano, 1996). In addition, according to attribution theory (Heider, 1958; Weiner, 1986), we reveal the moderating effect of customer blame attribution on the relationship between customer incivility and employees’ emotions. A two-wave and two-source data was used to test our theoretical model.

This research makes three contributions. First, we enrich the literature on customer incivility by taking the lead in exploring the positive impact of customer incivility and clarifying its double-edged effect. The exploration of the positive results of customer incivility challenges the previous research that customer incivility can only bring negative results (e.g., Al-Hawari et al., 2020; Cheng et al., 2020a). Second, this research contributes to affective events theory by revealing the mediating roles of hostility and guilt. These two discrete emotions illustrate employees’ psychological reactions to customer incivility and provide the evidence to explain why different employees take different behaviors. Third, our study extents the research on blame attribution by exploring the moderating effect of customer blame attribution. We deepened the understanding of under what conditions which emotion arise by integrating employees’ emotions and attributions.

## Theories and hypotheses

### Affective events theory

Affective events theory posits that the relevance, nature, and meaning of specific work events prompt individuals to experience corresponding proximal emotions (Weiss and Cropanzano, 1996). Hassles, or negative events, hinder the achievement of work goals and are associated with negative emotions; while uplifts, or exciting events, promote the realization of work goals and are closely related to positive emotions (Weiss and Cropanzano, 1996; Carnevale et al., 2021). “Meaning analysis” of events induces specific discrete emotions (Weiss and Cropanzano, 1996, p. 33). This process is influenced by personal characteristics (Weiss and Cropanzano, 1996; Ganegoda and Bordia, 2019). Subsequently, emotions drive individuals to take actions to response (Weiss and Cropanzano, 1996).

Customer incivility can be interpreted as a stress event that hinders the achievement of employees’ work goals and elicits negative emotions. After analyzing the meaning of the event, hostility arises if employees believes customers are against themselves (Chi et al., 2013), which leads to their revenge behavior. If employees perceive that customers are aiming at the actual service quality, that is, they fail to achieve their work goals, they will feel guilty. In order to make amends, employees will perform customer-oriented behavior.

### Attribution theory

Attribution theory postulates that individuals have an innate need to interpret events and find out the reasons, especially when events are unexpected (Weiner, 1986). Any attribution should constitute a reasonable explanation of what happened, so as to influence individuals’ behavioral response to these outcomes (Heider, 1958; Tomlinson and Mayer, 2016). An important role of attribution is to indicate who or what is to blame for the outcome (Heider, 1958).

**FIGURE 1:**
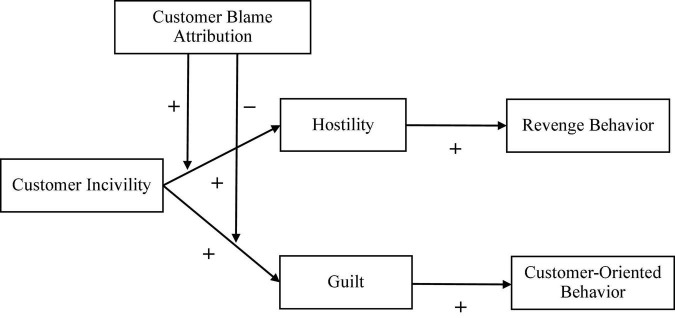
Theoretical model.

In general, individuals tend to take responsibility for positive outcomes and are eager to find scapegoats and blame others for negative outcomes (Bowman, 1978). When faced with unfriendly customers, employees are very likely to blame customers (i.e., customer blame attribution). Therefore, we argue that customer blame attribution can, on the one hand, strengthen employees’ hostility toward customers; on the other hand, weaken employees’ feelings of guilt. See Figure 1 for theoretical model.

### Customer incivility as an affective event and predictor of employees’ hostility and guilt

Customer incivility stems from workplace incivility, which is defined by Andersson and Pearson (1999) as a “low-intensity deviant behavior with ambiguous intent to harm the target, in violation of workplace norms for mutual respect” (p. 457). More and more literature on customer incivility shows that customers are a unique and important source of uncivilized interaction in work (Sliter et al., 2012; Liu et al., 2019). Unlike the interactions between members of the organization, since customer service work is often a one-time contact between employees and customers (especially in the restaurant and hospitality industries), there is no common past experience between them and they are unlikely to interact again in the future (Grandey et al., 2004; Wilson and Holmvall, 2013). If employees lack discretion and autonomy, they cannot reasonably solve the problems in the face of customers’ demands. And customers often do not suffer punishment when they treat employees unfriendly. In this way, any dissatisfaction of customers can be expressed and vented by low-intensity incivility behavior to frontline employees. The cause of dissatisfaction may have nothing to do with employees (they become scapegoats), this results in more frequent customer incivility. Therefore, scholars generally claim that customer incivility is a kind of event with high frequency and low degree, including verbal insults, foul language and slight physical aggression by customers (Sliter et al., 2012; Walker et al., 2014; Shin and Hur, 2022).

According to affective events theory, customer incivility, as a kind of typical troublesome and negative event, can give rise to negative emotions of employees (Weiss and Cropanzano, 1996). In this study, customer incivility is regarded as a kind of affective events. These events imply that the interaction between frontline employees and customers is inharmonious, which hinders the successful realization of employees’ work goals, and also endangers the daily operation of the enterprise. When these events occur, customers “violate social norms of mutual respect and courtesy in service exchanges” (Walker et al., 2014, p. 152). With the continuous accumulation of customer incivility events, when they reach a certain level, they will stimulate employees’ negative emotions (Cheng et al., 2020b; Shin and Hur, 2022).

Affective events theory postulates that individuals differ in their “meaning analysis” of events, resulting in different specific discrete emotions (Weiss and Cropanzano, 1996). This research proposes that customer incivility can cause two different kinds of discrete negative emotions of employees: hostility and guilt.

Hostility is a negative emotion typically triggered by others’ behavior, an outward-focused emotion that arises when someone or something gets in the way of one’s goals, or when an individual believes that another person is hurting him or herself in some way or inflicting physical or psychological pain (Livne-Ofer et al., 2019). As a stressor for frontline employees, customer incivility events are destructive and give employees painful experiences. So that employees are likely to interpret them as provocations to their own work (Chi et al., 2013). Provocations means interfering with and frustrating employees’ work goals (Liang et al., 2016). In this regard, perceived interpersonal provocation is a prerequisite for experienced hostility by the individual toward the provocateur, and the most typical and immediate response to provocation is to experience hostility (Mayer et al., 2012; Liang et al., 2016). Therefore, when confronted with someone who provokes them, employees become strongly emotionally inflamed and hostile to the provocateur (Liang et al., 2016). When customer incivility events occur, employees may view customers as “abusers” and as external obstacles to the work. At this time, employees may think that they are the hostile targets of customers, which will lead to hostility. Thus, we propose:

*Hypothesis 1a: Customer incivility is positively related to employees’ hostility.*

Unlike hostility, guilt is a self-conscious, inward-focused emotion caused by self-evaluation and self-reflection (Tangney et al., 2007; Livne-Ofer et al., 2019), and is an unpleasant and remorseful feeling related to admitting that one has violated moral or social standards (Tangney et al., 2007; Livingston and Judge, 2008). This feeling may be triggered by behaviors that violate social norms, or by failure to prevent negative behaviors (Tracy and Robins, 2006).

In the process of interaction with customers, employees are not completely equal to customers due to their occupational requirements. Service employees bear “a primary ethical responsibility” of providing customers with “good quality products and services” (Kaptein, 2008, p. 982). Customer incivility is a typical event that violates social norms (Walker et al., 2014), and regardless of the cause, it is usually interpreted by employees as a catharsis of dissatisfaction and can lead to guilt if employees reflect themselves from an internal perspective. This guilt is manifested in two aspects: first, employees are likely to feel guilty toward customers for not being able to meet customers’ needs (whether they are reasonable or not), or for not being perfect (i.e., service failure, Baumeister et al., 1994). Second, job responsibilities (i.e., duty violation) can also make employees feel guilty for customer incivility. The enterprise has the responsibility to control its business premises (service space) to maintain normal business activities (Boukis et al., 2020). Therefore, as the executor of enterprise activities, employees are also responsible for orderly management of service space. When customer incivility events occur, it means that orderly business activities are challenged, and employees have lost control of the service space. When employees can observe customer incivility and think the matter (which should have been under their control) is out of their control, that is, they fail to prevent the occurrence of customers’ incivility, they will feel guilt toward the company. Significantly, even if the customer is the cause, they still feel guilty. This is because the professional responsibility (orderly operation) given to employees by the company at this time is not perfectly achieved. That is, they failed to achieve the goals of the organization (Tracy and Robins, 2006; Tangney et al., 2007). Hence, we propose:

*Hypothesis 1b: Customer incivility is positively related to employees’ guilt.*

### The effect of employees’ hostility and guilt on behavioral response

Affective events theory states that different emotions urge individuals to adopt different behaviors (Weiss and Cropanzano, 1996). It is widely believed that positive emotions expand individuals’ thinking and lead them to take positive actions, while negative emotions narrow individuals’ thinking and lead them to take negative actions (Fredrickson, 1998). However, some recent studies have shown that some positive emotions narrow the thinking, while some discrete negative emotions broaden it (Harmon-Jones et al., 2013; Becker and Curhan, 2018).

In this research, we argue that hostility (an outward-focused emotion) will narrow employees’ thinking and trigger them to perform revenge behavior toward customers. Conversely, guilt (an inward-focused emotion) will expand employees’ thinking and elicit their customer-oriented behavior.

Hostility is associated with threats to individual self-esteem and goals (Mayer et al., 2012). Hostility is aversive, promotes the desire of individuals to take radical actions, and makes individuals act toward the source of emotion (i.e., the provocateur) (Liang et al., 2016; Chen et al., 2021). Therefore, we presume that when employees have high hostility toward customers, they tend to vent their emotions through impulsive behaviors (Livne-Ofer et al., 2019), such as lashing out and aggressing against customers.

Revenge behavior is exactly what individuals with hostile emotions needs. Revenge behavior refers to the infliction of harm in return for perceived wrong (Bradfield and Aquino, 1999; Aquino et al., 2001). This behavior can help individuals restore self-esteem (Bradfield and Aquino, 1999). A stream of research has confirmed that hostility leads to individuals’ revenge (e.g., Mayer et al., 2012; Livne-Ofer et al., 2019; Cheng et al., 2020b). Therefore, we assume that employees’ hostility will perform the revenge behavior toward unfriendly customer.

*Hypothesis 2: Employees’ hostility is positively related to revenge behavior.*

When individuals have guilt feelings toward others, they feel negatively evaluated and engage in self-reflection (Livne-Ofer et al., 2019). This emotion reflects the discrepancy between the ideal self and the actual self (Livne-Ofer et al., 2019), that is, the failure to achieve personal goals. To compensate for guilt toward others, or to achieve their personal goals, guilty individuals usually develop constructive intentions and subsequent constructive or reparative behaviors (Tangney et al., 2007).

We consider customer-oriented behavior to be a typically constructive behavior. Customer-oriented behavior refers to employee behavior that focuses on meeting customer needs and engendering customers satisfaction (Grizzle et al., 2009). Guilty employees will try their best to think from customers’ perspective, value customers’ experience, and satisfy customers as much as possible, and therefore will act constructive or reparative behaviors toward customers (Tangney et al., 1998). Thus, we assume that employees’ guilt will perform customer-oriented behavior.

*Hypothesis 3: Employees’ guilt is positively related to customer-oriented behavior.*

Affective events theory and many empirical evidences demonstrate that emotions play a mediating role between work events and individual behavior (Weiss and Cropanzano, 1996; Elfenbein, 2007; Carnevale et al., 2021). Customer incivility events threaten employees’ goal of successfully completing their work. At this time, customers are regarded as provocateurs by employees, thus employees will develop a hostile emotion that drives them to take radical or aggressive action against customers, thereby alleviating the dislike for customers (Liang et al., 2016). Therefore, hostile emotions will eventually drive employees to take revenge (Mayer et al., 2012; Liang et al., 2016).

On the other hand, customer incivility events demonstrate that customers are dissatisfied with the service provided by employees. Thus, feelings of guilt toward customers and the organization arise as a result of employees’ failure to stop customer incivility and to successfully achieve the goal of serving customers well (Tracy and Robins, 2006; Tangney et al., 2007; Livne-Ofer et al., 2019). Therefore, in order to make amends and successfully achieve the work goals, employees who feel guilty will take reparative measures and implement customer-oriented behaviors (Tangney et al., 1998). Thus, we propose:

*Hypothesis 4a: Employees’ hostility mediates the positive effect of customer incivility on revenge behavior.*

*Hypothesis 4b: Employees’ guilt mediates the positive effect of customer incivility on customer-oriented behavior.*

### The moderating effect of customer blame attribution

Attribution theory claims that individuals often try to understand the surrounding environment by making attributions about the causes of events, which in turn will affect individual cognition, emotion and future behavior (Weiner, 1986; Tomlinson and Mayer, 2016). When faced with important events, individuals tend to find out who is responsible for them (Heider, 1958). The happening of customer incivility events means the occurrence of service scenarios conflicts, and also means that the service goals of employees and enterprises are being threatened. Therefore, we believe that in the face of such negative events, employees are eager to know who is to blame (Martinko et al., 2007).

Customer blame attribution refers to that the employee blames the customer for the events and thinks that the customer should bear primarily responsible (Bradfield and Aquino, 1999). There is a general tendency for individuals to attribute the success of events to themselves and to blame others for the faults of events (Heider, 1958; Bowman, 1978). Thereby, we argue that when confronted with customer incivility, it is highly likely that employees will attribute fault to the customer, creating customer blame attribution.

As mentioned earlier, customer incivility can lead to employees’ hostility and thus revenge behavior. Customer blame attribution are formed when employees believe that customers incivility is hurting them and that this behavior is unnecessary (e.g., the customers could have reacted in a different way) (Garcia et al., 2019). Moreover, in the case of high customer blame attribution, customers are not only considered to violate interpersonal norms, but their behavior is also perceived as deliberate, unwarranted, and provocative (Mikula, 2003; Garcia et al., 2019). As a result, employees develop more severe hostile feelings toward customers, which in turn leads to more frequent revenge behavior.

In contrast, when customer incivility triggers employees’ guilt, if employees believes that the responsibility for the incivility lies with customers rather than them, the intensity of their own guilt will be weak, which in turn will reduce the frequency of customer-oriented behavior. This is the premise that the generation of employees’ guilt is based on their failure to serve the customers well. If customers’ incivility is intentional, or customers’ requests are far beyond employees’ ability, customer blame attribution will reduce the likelihood of employee self-reflection (Tangney et al., 2007; Livne-Ofer et al., 2019), thus reducing employee guilt and the frequency of performing customer-oriented behavior. In other words, compared with the employee with a low level of customer blame attribution, the employee with a high level of customer blame attribution will have a lower degree of guilt when facing the same degree of customer incivility, and will perform less frequent customer-oriented behavior. We propose the following hypotheses:

*Hypothesis 5a: Customer blame attribution moderates the positive indirect effect of customer incivility on revenge behavior via employees’ hostility, such that the indirect effect is stronger when customer blame attribution is high (vs. low).*

*Hypothesis 5b: Customer blame attribution moderates the positive indirect effect of customer incivility on customer-oriented behavior via employees’ guilt, such that the indirect effect is weaker when customer blame attribution is high (vs. low).*

## Materials and methods

### Samples and procedures

Our study was conducted at a large chain restaurant company in Beijing, China. This company is famous for its unique dishes and enthusiastic service. For example, if a customer comes here for consumption on his/her birthday, the restaurant will prepare a birthday cake for this customer, and the service personnel will sing birthday songs to congratulate him/her warmly. Through interviews with frontline service employees and company managers, we found that in this company, customer incivility events are frequent, so this company is an ideal research target. The company’s human resources (HR) department assisted us in randomly selecting 460 frontline service employees and their direct leaders (53 supervisors). The research team screened participants to ensure that they had all experienced at least one unpleasant event in the past 2 weeks that made them feel uncomfortable. We informed all participants of the purpose of this survey, and guaranteed the confidentiality and anonymity of the survey and explained the specific matters to be taken. Once the questionnaires were completed, they were sealed in envelopes and submitted to the research team.

Past studies have demonstrated that a 2-week interval can better measure variables (e.g., Bani-Melhem, 2020), which can minimize potential common method bias and reduce participant fatigue (Podsakoff et al., 2003). To avoid bias in the measurement of behavior due to loss of judgment in individuals immersed in emotions, we measured emotion and behavior separately. Therefore, we executed a two-source, two-wave survey to collect data. Specifically, the Time 1 survey collected data on employees’ demographics, customer incivility, customer blame attribution, hostility, and guilt. 417 employees completed this round survey. The Time 2 survey collected data on employees’ revenge behavior, and customer-oriented behavior (this variable reported by supervisors). In this round, 383 employees and 45 supervisors completed the survey.

Lastly, after deleting all missing and invalid data, our final sample consisted of 366 matched employee-supervisor pairs with an overall response rate of 79.6%. Of these frontline service employees, 228 (62.3%) were female and 138 (37.7%) were male. 127 (34.7%) of them had a high school diploma or less, 108 (29.5%) of them had a junior college degree, and 131 (35.8%) had a bachelor’s degree or above. Their average age was 34.48 years (*SD* = 7.956), organizational tenure was 6.22 years (*SD* = 3.520).

### Measures

Unless otherwise specified, all items were measured on a 5-point Likert-type scale using established scales. In order to ensure that the measurement tools are suitable for Chinese situations, we strictly followed Brislin’s (1986) standard translation and back-translation procedures which guarantees the equivalence of item meaning. And all items were present in Mandarin Chinese.

### Customer incivility

We measured customer incivility using a four-item scale (Cronbach’s α = 0.966) developed by Walker et al. (2014). This scale ranged from 1 to 5 (1 = *never*, 2 = *once in the interaction*, 3 = *a few times in the interaction*, 4 = *in most of the interaction*, and 5 = *in all of the interaction*). The question stem was “In the past 2 weeks, how frequently have you experienced the following events?” A sample item reads, “Customers spoke aggressively toward me”.

### Customer blame attribution

We captured customer blame attribution *via* four-item scale (Cronbach’s α = 0.885) adapted from Bradfield and Aquino’s (1999) scale. Items include: “I blamed these customers,” “These customers wronged me,” “I was victimized,” and “These customers are guilty.” This scale ranged from 1 (*completely disagree*) to 5 (*completely agree*).

### Hostility

Following Chen et al. (2021), we assessed hostility using six items (Cronbach’s α = 0.910) from the Positive and Negative Affect Schedule (Watson and Clark, 1999). This scale was measured immediately after the Customer Incivility Scale. The question stem was “To what extent do you feel the following emotions when above events happen to you?” These items were “hostile,” “disgusted,” “irritable,” “angry,” “scornful,” and “loathing,” all ranged from 1 (*very slightly*) to 5 (*very strongly*).

### Guilt

Consistent with Livingston and Judge (2008), we captured employees’ guilt using a three-item scale (Cronbach’s α = 0.943) developed by Izard et al. (1974). The question stem and measurement method were the same as that of the Hostility scale. These three items were “repentant,” “guilty,” and “blameworthy.”

### Revenge behavior

We used a six-item scale (Cronbach’s α = 0.962) from Bradfield and Aquino (1999) to capture employees’ revenge behavior. This scale ranged from 1 (*never behave this way*) to 5 (*always behave this way*). The question stem was “In the last 2 weeks, how frequently have you performed the following behaviors when you interact with impolite customers?” An example item is “I tried to make something bad happen to them.”

### Customer-oriented behavior

We adapted Grizzle et al. (2009) seven-item scale (Cronbach’s α = 0.900) to assess customer-oriented behavior. In order to reduce the participants’ self-serving bias, all items were rated by their supervisors. This scale ranged from 1 (*never behave this way*) to 5 (*always behave this way*). The question stem was “In the last 2 weeks, how frequently have this employee performed the following behaviors when he/she interacts with customers?” An example item is “[This employee] gave courteous service to customers.”

### Control variables

Following similar literature (e.g., Walker et al., 2014; Al-Hawari et al., 2020), in this study, we controlled for employees’ age, gender (0 = female, 1 = male), education (1 = high school diploma or less, 2 = junior college degree, 3 = bachelor’s degree or above), and organizational tenure.

## Results

### Confirmatory factor analysis

Confirmatory factor analysis was implemented to examine the convergence validity and discriminant validity of the theoretical variables by using Amos 23.0. The results revealed that the six-factor model fit neatly into the data (χ^2^ = 734.393, *df* = 390, CFI = 0.968, GFI = 0.885, IFI = 0.968, TLI = 0.964, RMR = 0.044, RMSEA = 0.049). As shown in Table 1, all factor loadings were larger than 0.6, the composite reliability (CR) of each variable exceeded 0.8, average variance extracted (AVE) by each variable exceeded 0.5, all of these illustrated that convergence validity was acceptable. Table 2 demonstrates that each variable’s discriminate validity value (square root of AVE) exceeded Pearson correlation value. Therefore, our measurement model exhibits acceptable values and validity.

**TABLE 1:** Results of confirmatory factor analysis.

Variables	Estimate	CR	AVE
1. Customer incivility	0.926–0.949	0.966	0.878
2. Customer blame attribution	0.779–0.852	0.885	0.659
3. Hostility	0.680–0.987	0.913	0.680
4. Guilt	0.907–0.935	0.943	0.847
5. Revenge behavior	0.780–0.998	0.966	0.828
6. Customer oriented behavior	0.712–0.794	0.901	0.566

CR, composite reliability; AVE, average variance extracted.

**TABLE 2:** Descriptive statistics and bivariate correlations among study variables.

Variables	1	2	3	4	5	6	7	8	9	10
1. Age	1									
2. Gender	–0.026	1								
3. Education	–0.02	0.07	1							
4. Organizational tenure	–0.067	0.069	–0.004	1						
5. Customer incivility	0.035	0.041	0.055	0.089	**0.937**					
6. Customer blame attribution	0.03	–0.061	–0.099	0.072	0.049	**0.812**				
7. Hostility	–0.071	0.034	0.025	0.146**	0.348**	0.130*	**0.825**			
8. Guilt	0.07	0.007	0.052	–0.026	0.520**	–0.025	0.013	**0.920**		
9. Revenge behavior	0.021	0.075	–0.006	0.072	0.150**	0.129*	0.360**	0.075	**0.910**	
10. Customer oriented behavior	0.035	–0.069	0.048	–0.037	0.051	0.058	0.011	0.195**	–0.019	**0.752**
Mean	34.480	0.380	2.010	6.221	2.865	2.986	2.680	3.074	2.691	3.025
SD	7.956	0.485	0.841	3.520	1.003	0.686	0.909	0.956	1.001	0.560

*N* = 366. *p < 0.05, **p < 0.01. Gender: 0 = female, 1 = male; Education: 1 = high school diploma or less, 2 = junior college degree, 3 = bachelor’s degree or above. Diagonal elements (in bold) are the square root of the average variance extracted (AVE).

### Descriptive statistics

Table 2 demonstrated the means, standard deviations, reliabilities, and correlations of all variables in this study. Customer incivility is positively associate with hostility (*r* = 0.348, *p* < 0.01) and guilty (*r* = 0.520, *p* < 0.01); hostility is positively associate with revenge behavior (*r* = 0.360, *p* < 0.01); and guilty is positively associate with customer-oriented behavior (*r* = 0.195, *p* < 0.01).

### Hypothesis testing

Conditional process analysis was conducted to test our hypotheses by using the PROCESS macro (Hayes, 2022) for SPSS 26.0. The results of path estimates are showed in Table 3. As demonstrated in Table 3, the positive effect of customer incivility on employees’ hostility was significant (β = 0.310, *SE* = 0.044, *p* < 0.01), and the positive effect of customer incivility on employees’ guilt was significant (β = 0.497, *SE* = 0.043, *p* < 0.01). Moreover, employees’ hostility was significantly and positively associated with their revenge behavior (β = 0.396, *SE* = 0.055, *p* < 0.01), employees’ guilt was significantly and positively associated with their customer-oriented behavior (β = 0.112, *SE* = 0.030, *p* < 0.01). In summary, all the above proved that Hypotheses 1a, 1b, 2, and 3 were supported by data.

**TABLE 3:** Conditional process analysis.

Variables	Hostility	Guilt	Revenge behavior	Customer oriented behavior
	**β (*SE)***	**β (*SE)***	**β (SE)**	**β (SE)**
Constant	2.770** (0.242)	2.951** (0.237)	1.376** (0.307)	2.633** (0.179)
Age	–0.009 (0.006)	0.006 (0.005)	0.006 (0.006)	0.001 (0.004)
Gender	0.017 (0.091)	–0.016 (0.089)	0.132 (0.102)	–0.082 (0.060)
Education	0.024 (0.052)	0.018 (0.051)	–0.022 (0.059)	0.029 (0.035)
Organizational tenure	0.027* (0.013)	–0.018 (0.012)	0.005 (0.014)	–0.004 (0.008)
Customer incivility	0.310** (0.044)	0.497** (0.043)		
Customer blame attribution	0.137* (0.064)	–0.058 (0.063)		
Hostility			0.396** (0.055)	
Guilt				0.112** (0.030)
Customer incivility × customer blame attribution	0.191** (0.060)	–0.127* (0.059)		
*R* ^2^	0.176	0.290	0.137	0.046
*F*	10.912**	20.872**	11.398**	3.478**
**Conditional indirect effects *via* hostility**	**Effect**	**Boot *SE***	**Boot LL 95% CI**	**Boot LL 95% CI**
Mean – 1 SD	0.071	0.033	0.013	0.141
Mean	0.123	0.033	0.063	0.194
Mean + 1 SD	0.175	0.047	0.088	0.273
**Conditional indirect effects *via* guilt**	**Effect**	**Boot *SE***	**Boot LL 95% CI**	**Boot UL 95% CI**
Mean – 1 SD	0.066	0.019	0.030	0.105
Mean	0.056	0.016	0.025	0.089
Mean + 1 SD	0.046	0.016	0.018	0.081

*N* = 366. *p < 0.05, **p < 0.01. All coefficients are unstandardized. Bootstrap sample size = 5,000. SE, standard error; LL, low limit; UL, upper limit; CI, confidence interval.

The conditional process analysis with a 5000-resample bootstrap method was conducted to test the mediating effects and the moderated mediation effects (Preacher et al., 2007). As shown in Table 3, the positive indirect effect of customer incivility on revenge behavior *via* employees’ hostility was significant [β = 0.123, *SE* = 0.033, 95% CI = (0.063, 0.194)], and the positive indirect effect of customer incivility on customer-oriented behavior *via* employees’ guilt was significant [β = 0.056, *SE* = 0.016, 95% CI = (0.025, 0.089)]. Thus, Hypothesis 4a and 4b were supported.

**FIGURE 2:**
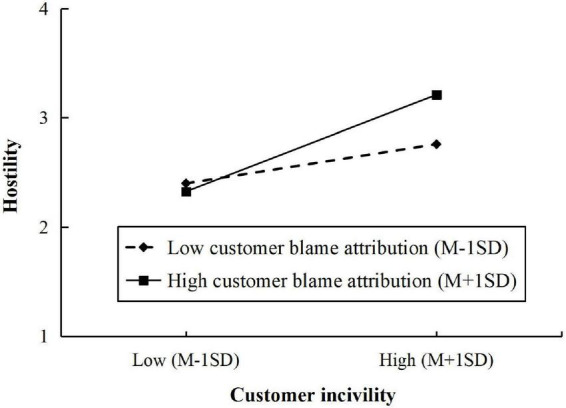
The moderating effect of customer blame attribution on the relationship between customer incivility and hostility.

**FIGURE 3:**
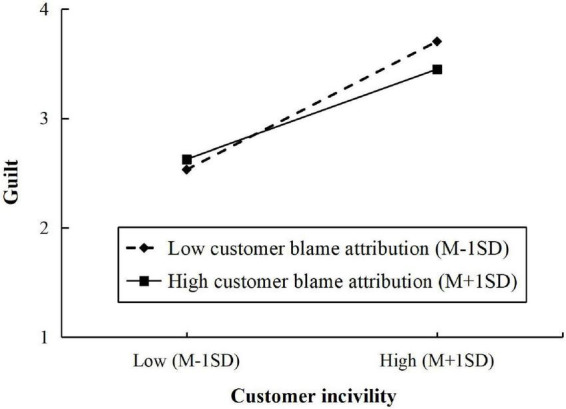
The moderating effect of customer blame attribution on the relationship between customer incivility and guilt.

The coefficient estimates of moderated mediation effects is presented in Table 3. The interaction effect of customer incivility and customer blame attribution on employees’ hostility was significant (β = 0.191, *SE* = 0.060, *p* < 0.01). As shown in Table 3 and Figure 2, the positive relationship between customer incivility and revenge behavior *via* employees’ hostility was strengthened when employees’ customer blame attribution was higher [β = 0.175, *SE* = 0.047, 95% CI = (0.088, 0.273)], compared with employees with low level of customer blame attribution [β = 0.071, *SE* = 0.033, 95% CI = (0.013, 0.141)]. In addition, a significant interaction effect of customer incivility and customer blame attribution on employees’ guilt was found (β = -0.127, *SE* = 0.059, *p* < 0.05). The relationship is presented in Figure 3. The positive indirect effect of customer incivility on customer-oriented behavior through employees’ guilt was significantly weaker when employees’ customer blame attribution was higher [β = 0.046, *SE* = 0.016, 95% CI = (0.018, 0.081)], compared with when employees’ customer blame attribution was lower [β = 0.066, *SE* = 0.019, 95% CI = (0.030, 0.105)]. Hence, Hypothesis 5a and 5b were supported.

## Discussion

Drawing on affective events theory and attribution theory, the current research proposed and examined a moderated dual-mediator causal model to explore how and when customer incivility leads to different employee behaviors by eliciting different discrete emotions from employees. Our findings show that, on the one hand, customer incivility can cause employees’ hostility toward customers, which in turn leads to revenge behavior, and customer blame attribution enhances the effect of customer incivility on hostility. On the other hand, customer incivility triggers employees’ guilt, which leads to customer-oriented behavior, yet customer blame attribution weakens this relationship.

### Implications for theory

The primary contribution of this study is to provide a more comprehensive understanding of the influence of customer incivility on employees’ behavior. The double-edged effect of customer incivility challenges the current mainstream view, that is, customer incivility can only cause negative outcomes (e.g., Sliter et al., 2012; Walker et al., 2014), and enriches the study of customer incivility. Our research makes up for the limitation of focusing only on the negative results of customer incivility in the past studies (e.g., Hur et al., 2015) by revealing the positive outcomes that customer incivility can trigger employees’ customer-oriented behavior. In summary, the revelation of the double-edged effect has led to a deeper and comprehensive understanding of the impact of customer incivility.

Second, we extent affective events theory by examining the mediating roles of hostility and guilt. Affective events theory points out that work events can impact employees’ behavior by influencing their emotions (Weiss and Cropanzano, 1996). This study is not limited to the extremely obvious emotion—hostility, but also reveals the relatively secret emotion—guilt. We link guilt to customer incivility and employees’ customer-oriented behavior, and explain why customer incivility leads to employee guilt based on service failure and duty violation. Our dual emotion perspective enriches affective event theory. In sum, we extend the specific application of affective events theory to explore the relationship between customer incivility, employee emotions, and behaviors.

Third, we enrich the research on attribution by focusing on customer blame attribution. The proposition of this moderating variable makes us strengthen our insight into how and when customer incivility will lead to employees’ hostility and guilt, respectively. In particular, customer blame attribution plays a different role in moderating the relationship between customer incivility and two discrete emotions. Meanwhile, customer blame attribution deepens our understanding of the relationship between customer incivility and emotions, and expands the study of boundary conditions (Bedi and Schat, 2017; Cheng et al., 2020b).

### Implications for practice

This research provides several managerial implications. First, our finding revealed that the double-edged effect of customer incivility. That is, customer incivility can cause both revenge behavior and customer-oriented behavior. To achieve organizational goals and serve customers as well as possible. Companies should select employees which are not prone to anger and hostility when hiring based on personality traits. Meanwhile, the necessary training is provided to reduce employees’ hostile reactions and enhance their sense of guilt.

Second, we found that blame attribution plays an important role in employees’ emotional reactions when faced with incivility. Companies should pay attention to the attributions of employees facing workplace emergencies and help them establish scientific and reasonable attributions. That is, minimize customer blame attribution of frontline service employees to avoid triggering hostile emotions that bring about aggressive behavior.

### Limitations and future research

Our research has several limitations. We discussed the emotional and behavioral reactions of employees when confronted with customer incivility. We expect future research to explore a third-person perspective, i.e., to explore the reactions of other customers who witness customer incivility events. In addition, our research object is offline service personnel, and online customer incivility may vary due to different situations (Bacile et al., 2018, 2020). We also expect that future research will extend the research perspective to the online environment.

Second, our study examines the impact of customer blame attribution on the model from an attribution perspective. We call for future research to enrich the inclusion of boundary variables. For example, organization adaptive practices responding to events (Lin et al., 2021), similarity of customers and employees, and gender (Bedi and Schat, 2017; Cheng et al., 2020b) can be added to explore in depth their possibility as moderating variables of customer incivility.

In addition, we adopted a two-source, two-wave method to measure emotion and behavior separately, which may lead to the weakening of correlation between variables to some extent. Future research could consider simultaneous measurement or experimental method. However, it should be noted that an emotional individual may cause measurement bias when evaluating their behaviors.

Finally, our sample was collected from Chinese. Chinese traditional culture emphasizes maintaining harmonious interpersonal relationships, highlights the service provider’s tolerance of the customer in service situations, and usually advocates avoiding conflict or aggressive behavior. This inhibits the negative effects of customer incivility to some extent. Therefore, future studies can collect data from more backgrounds and cultures to enhance the generalizability of our findings.

## Data availability statement

The raw data supporting the conclusions of this article will be made available by the authors, without undue reservation.

## Ethics statement

The studies involving human participants were reviewed and approved by the Ethical Review Board of Beijing Jiaotong University. Written informed consent for participation was not required for this study in accordance with the national legislation and the institutional requirements.

## Author contributions

CC made substantial contributions to the conception of the work and wrote the manuscript. MZ revised it critically for important intellectual content. YZ made contributions to the analysis of data for work. All authors contributed to the article and approved the submitted version.
